# Interaction analysis of monoaminergic polymorphisms and childhood environment related to personality functioning in patients with Borderline Personality Disorder

**DOI:** 10.1192/j.eurpsy.2024.1220

**Published:** 2024-08-27

**Authors:** E. Salgo, Z. Nemoda, E. Kenézlői, E. Lévay, L. Balogh, B. Bajzát, J. Réthelyi, Z. S. Unoka

**Affiliations:** Semmelweis University, Budapest, Hungary

## Abstract

**Introduction:**

Neurobiological studies have shown that genetic variations affecting the intensity of monoamine neurotransmission play an important role in aggressive behavior and borderline personality traits. Also, the effect of family environment has been repeatedly shown on aggressive behavior and interpersonal functioning. Population-based longitudinal studies pointed out interactions between the so-called monoaminergic sensitivity alleles and childhood adversities.

**Objectives:**

Our study aimed to analyze the associations between the most studied variable number tandem repeats of monoaminergic genes and the different psychological factors in adult patient and healthy control groups, checking for the moderating effects of the parental occupation and education, childhood abuse and trauma.

**Methods:**

The recruited 73 patients with BPD diagnosis and 98 healthy controls were assessed by the Structured Clinical Interview for DSM-5. Participants filled out online questionnaires including the Level of Personality Functioning Scale – short version (LPFS-SR) and the Buss-Perry Aggression Questionnaire (BPQ). Childhood social environment and traumatic experiences were assessed by the Barratt Simplified Measure of Social Status and the Early Trauma Inventory or the Childhood Trauma Questionnaire. Genomic DNA samples were obtained either from peripheral blood, saliva or buccal swabs using the desalting technique. Functional dopaminergic and serotonergic polymorphisms were chosen based on previous findings, implicating them as sensitivity gene variants, e.g., the variable-number tandem-repeats of the dopamine D4 receptor, serotonin transporter and the monoamine oxidase-A (MAO-A) genes. Since the MAO-A gene is located on the X chromosome, sex-stratified analyses were also carried out.

**Results:**

Family environment indexed by the Barratt Simplified Measure Social Status had significant effect on anger, hostility and interpersonal functioning (p < 0.01). In the pooled sample of patients and controls, individuals carrying the high activity alleles of MAOA had elevated scores on the BPQ subscales. When analysis was limited to female participants, the genetic effect stayed significant only at the anger scale of the BPQ.

**Conclusions:**

Family environment had pronounced effect on aggressive behavior and personality functioning, interaction with common monoaminergic genetic variants was detected only in women.

*This study was supported by the National Research Development and Innovation Office grants NKFI K 129195 and NKFI K 135437.*

**Disclosure of Interest:**

None Declared

